# Ubiquitous Green Computing Techniques for High Demand Applications in Smart Environments

**DOI:** 10.3390/s120810659

**Published:** 2012-08-03

**Authors:** Marina Zapater, Cesar Sanchez, Jose L. Ayala, Jose M. Moya, José L. Risco-Martín

**Affiliations:** 1 CEI Campus Moncloa, UCM-UPM, Madrid 28040, Spain; 2 Electronic Engineering Department, ETSI Telecomunicación, Universidad Politécnica de Madrid, Madrid 28040, Spain; E-Mail: josem@die.upm.es; 3 IMDEA Software Institute, and Institute for Applied Physics, CSIC, Madrid 28660, Spain; E-Mail: cesar.sanchez@imdea.org; 4 DACYA, Universidad Complutense de Madrid, Madrid 28040, Spain; E-Mails: jayala@fdi.ucm.es (J.L.A.); jlrisco@dacya.ucm.es (J.L.R.-M.)

**Keywords:** ubiquitous sensor network, green computing, heterogeneous systems, data centers, high performance computing, smart cities, ambient intelligence

## Abstract

Ubiquitous sensor network deployments, such as the ones found in Smart cities and Ambient intelligence applications, require constantly increasing high computational demands in order to process data and offer services to users. The nature of these applications imply the usage of data centers. Research has paid much attention to the energy consumption of the sensor nodes in WSNs infrastructures. However, supercomputing facilities are the ones presenting a higher economic and environmental impact due to their very high power consumption. The latter problem, however, has been disregarded in the field of smart environment services. This paper proposes an energy-minimization workload assignment technique, based on heterogeneity and application-awareness, that redistributes low-demand computational tasks from high-performance facilities to idle nodes with low and medium resources in the WSN infrastructure. These non-optimal allocation policies reduce the energy consumed by the whole infrastructure and the total execution time.

## 1. Introduction

In the last years, Ambient Intelligence has experienced a significant development, mainly because of the advances in the miniaturization of processors and the proliferation of embedded systems in many different objects and applications (e.g., communications, industrial, automotive, defense and healthcare environments). The International Data Corporation (IDC) Semiconductor research team announced in its April 2011 preliminary report that smart systems would consume more than 12.5 billion processor cores representing more than $100 billion in revenue by 2015 [1]. This means that there will be over six times more microprocessor cores in smart systems than in PCs.

The ambient intelligence paradigm was supported by these advances. Built mainly upon pervasive and ubiquitous computing, sensor networks and artificial intelligence, ambient intelligence has always had the goal of creating smart environments that aided people in their daily lives, reacting and adapting to their needs in an unnoticed way [2]. The information and knowledge society, as well as the Internet of Things, is surrounding peoples' lives with technology by the day, especially in urban areas. In 2008, about 50% of the world's population lived in urban areas, whereas it is envisaged that a 70% will live in cities by 2050 [3]. In this urban age cities are seen as places of prosperity and opportunities. Its citizens have high expectations that represent a challenge for the cities, especially from the technological point of view. Cities thus become the best candidate scenarios to develop the new technological challenges, giving birth to the concept of Smart Cities.

Smart Cities are cities that perform well on 6 characteristics: economy, people, governance, mobility, environment and living [4]. The goal of Smart Cities is to improve the way in which people interact with each other and with the urban environment. Their management represents a challenge in the field of Ubiquitous Sensor Networks (USNs), computing and communications. Smart Cities can be understood as a macro-scale case of the Ambient Intelligence paradigm, building upon the same principles and with the goal of offering a broad range of services, mainly in the framework of the so-called Internet of Services (IoS). They offer a broad number of services, ranging from intelligent buildings to tourism recommendations or tracking and monitoring systems. Because of the growth in population aging, one of the most common applications is healthcare, especially in the field of Ambient Assisted Living systems that provide services to elderly people [5]. The deployment of this kind of applications in a Smart City requires the usage of a large number of heterogeneous sensors (in the order of tens of thousands of units). They usually have in common the specifications that apply to Wireless Sensor Networks (WSNs): the need to be battery-powered, low power consumption, limited resources and small size.

Research has mainly paid attention to the communication and security challenges, as well as to the energy consumption of the Ubiquitous Sensor Networks. However, the Internet of Services has to deal with yet another computer paradigm: the High Performance Computing (HPC) infrastructures needed to tackle the computing demands in order to offer these services to the citizens. As all the data collected by the different sensors nodes has to be processed, converted and matched with other data so as to generate useful information, the computational needs of these systems are huge. Because of the nature of their workloads (the kind of processing that must be performed), this demand is usually satisfied by data centers.

Large data centers are composed of tens of thousands of servers with tens of peta bytes of storage, and multiple hundreds of giga bit bandwidth to the Internet. The electric bill of the data centers (including the electricity needed for cooling and air conditioning in the data center) was projected to pass 7 billion US dollars in the US alone, while the power density reached 60 *kW/m*^2^ for data centers by 2010. The Environmental Protection Agency (EPA), in its August 2007 report to the US Congress, affirmed that data centers consumed about 61 billion kilowatt-hours (kWh) in 2006, roughly 1.5 percent of total U.S. electricity consumption, for a total electricity cost of about $4.5 billion [6]. The EPA report also stated that the energy consumption of servers and data centers has doubled from 2002 to 2005. This rapid rates of growth in data center electricity use slowed significantly from 2005 to 2010 [7], but it still represents 1.3% of all the electricity use for the world, and 2% of all electricity use for the US, yielding worldwide to 250 billion kWh per year. According to a 2008 Gartner report [8], 50% of data centers would soon have insufficient power and cooling capacity to meet the demands of high-density equipment. Nowadays, apart from data center energy consumption and associated costs, there is an increasing interest in the environmental impact of data centers, in terms of their carbon dioxide (CO_2_) footprint.

Until now, it has been assumed that the computation needed by the applications of the Internet of Services would be performed “in the cloud”, without paying much attention to the inherent problems of this assumption. Only recent works have begun to work on architectures that take into account the computational needs [9]. This kind of approaches often disregard the energy consumption derived from the computation and cooling in the HPC facilities. However, the high economical and environmental impact of the energy consumption in data centers requires aggressive energy optimization policies. These policies have been already detected but not successfully proposed.

Our research work proposes an energy management solution to tackle the computational needs of smart environments and ambient intelligence applications that makes use of energy-minimization workload assignment policies. The goal is to minimize the energy consumption (and thus, the electricity bill) of the data centers that process the data provided by the sensors, by redistributing part of the computational demand on the HPC servers to the idle resources of a WSN infrastructure. This WSN infrastructure will be comprised of (i) a large amount of sensor nodes (in the order of thousands) with very-low resources; (ii) a smaller number (in the order of hundreds) of base stations with low resources and (iii) a few gateway nodes (the order of tens) with medium resources. The proposed solution off-loads computation from the HPC facility to the base station and gateway nodes, taking profit from their idle computing times. It does so by profiling the different tasks of the workload to be executed, classifying them in the HPC facility and predicting their energy parameters for the other nodes. The solution is thus based on application-awareness and node heterogeneity, and is built upon previous work, which proves that the usage of node heterogeneity at the data center level can yield substantial energy reductions [10]. In this case, however, the concept of heterogeneity is ported from the data center level to the smart service infrastructure. The main idea behind this work is to reduce energy by allocating the lowest-demand tasks to the low-power and low-resource nodes, while sending the highest-demand tasks to the HPC servers. This way, the workload is scheduled in the devices where it has a better performance. These techniques reduce considerably the amount of energy used by the High-Performance infrastructure while increasing performance. This work advances in the technology of energy-efficient computing in IoS and Ambient Intelligence applications, and in the mechanisms to place data centers in a more scalable and sustainable energy-efficiency curve.

This paper is organized as follows: Section 2 gives further information on the motivation and the related work on this topic. Section 3 presents an overview of the proposed solution. The allocation algorithms are presented in Section 4. Results and evaluation are shown in Section 5. Finally, the main conclusions of the paper are drawn in Section 6.

## 2. Related Work

A Smart City can be defined as a city that “uses information and communications technologies to make the critical infrastructure components and services of a city more aware, interactive and efficient” [11]. There is an important and bidirectional relationship between the Internet of Things, Services and People—that is, the future Internet—and the applications for Smart Cities and Ambient Intelligence. All of them need to provide support for heterogeneity, mobility, scalability, security, privacy and trust. Because of these similarities in functionality, they face the same challenges. Amongst other, some of these challenges are: (i) the need to manage heterogeneity in a large number of dispersed sensors and servers; (ii) the huge amount of heterogeneous information to be processed; (iii) the need to have this information available everywhere and always and (iv) and the need to use a common communication network.

**Figure 1.**
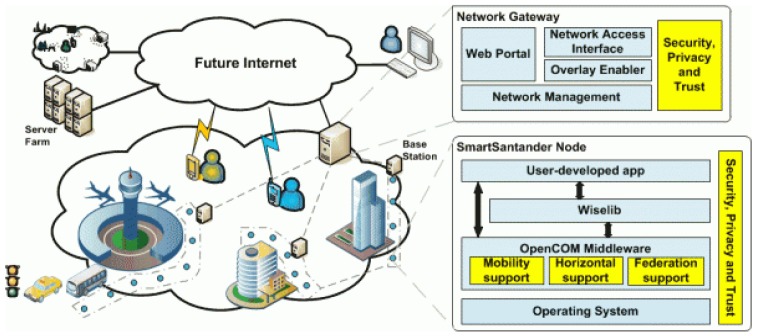
SmartSantander system architecture overview. Taken from [13].

Several European projects such as WISEBED [12] or SmartSantander [13] propose architectures (see Figure 1) over which all the communications and services of the Smart Cities can be built. They envision network deployments of more than 20,000 sensors. These works however propose only a high-level approximation that does not tackle the problem of the computational demand generated by this huge WSN.

One of the first problems regarding computational demand can be found in the nodes that are part of Ubiquitous Sensor Networks. These nodes have too few resources to satisfy the needs of the multiple monitoring algorithms needed to offer the user-demanded services. Moreover, the architectures of these nodes have been developed for a specific application, while the near future envisions nodes that should be able to collect information from very different natures. Also, the ever-changing world that face WSNs requires the development of applications based on evolutionary algorithms, artificial intelligence and learning. The power consumption of WSNs is not ready either to tackle with the computational loads needed for the algorithms and still be low-power and battery-operated.

The amount of information generated by a global deployment of a WSN thus implies the usage of a data center in order to store and process the obtained data. Meanwhile, these data center will have to provide the required infrastructure to perform all the computations needed by the executing applications. These applications are very different in nature, ranging from medical diagnosis applications to weather forecasts.

On the other hand, supercomputing facilities present a huge economic and environmental impact due to their very high power consumption. There are a number of different techniques to reduce the energy cost and power density in data centers in different levels of granularity: chip-level, server level, rack level, data center level, *etc.* Over the last years, this problem has been addressed by the well-known technique of Dynamic Voltage and Frequency Scaling (DVFS) [14], load monitoring [15], the introduction of heuristics to minimize the total power of a data center [16] or dynamic resource provisioning [17].

**Figure 2.**
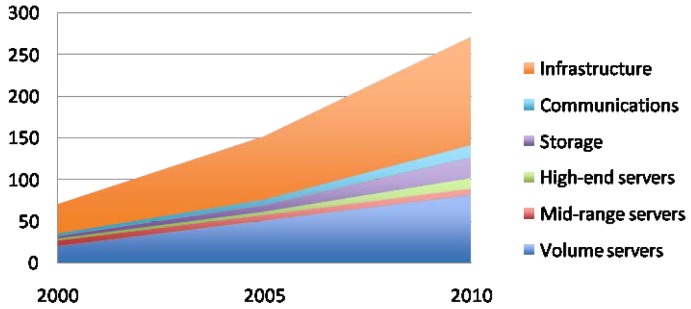
World servers installed (thousands).

**Figure 3.**
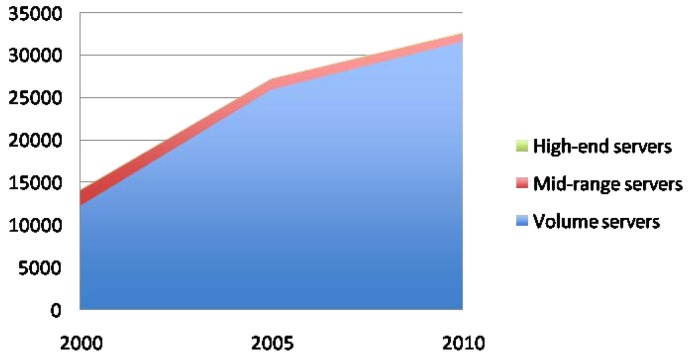
Electricity use by data centers (billion kWh/year).

In spite of all these measures, the energy consumption of data centers keeps growing, mainly because of the dramatic increase of supercomputing facilities. Figure 2 shows the number of world servers installed from 2000 to 2010. As it can be seen, the increase is currently reaching 5.75 million new servers per year. This translates into huge amounts of power consumption, mainly devoted to the infrastructure of the data centers and the volume servers (see Figure 3. The total energy usage by data centers is expected to exceed 400 GWh/year on 2015. Bearing this data in mind, it is clear that current effort is not enough, and that further research should be made to reduce power consumption of supercomputing facilities.

For data centers that have highly-variable loads, a very common technique for energy reduction is to move tasks from under-saturated servers to other servers and turn off the unused machines [18]. As the idle power of a server sometimes accounts for more that 50% of the maximum power of the server [19], great saving can come from using only the appropriate number of servers and turning off the unused ones. This is not the case, however, of data centers for smart-environment services. In HPC data centers with very stable workloads, occupancy levels are constantly high. This kind of data centers are usually dimensioned for the particular workload and the services they are using. These solutions would be complementary to our work and could be applied once some of the computation of the data center has been off-loaded to the WSN infrastructure—thanks to our allocation algorithms—and, thus, the occupancy levels become lower.

Most of the works proposing allocation algorithms have traditionally applied Mixed-Integer Linear Programming (MILP) or Mixed Integer Non-Linear Programming (MINLP) [20], Greedy algorithms [21] or Markov Chain algorithms [22] in order to generate the best task allocations. Most of these approaches do not propose a precise objective function and/or accurate mathematical formulation of the optimization problem. Although some of these solutions behave well in homogeneous data center level scenarios, they do not consider the heterogeneity inherent to smart environment applications. In this paper we consider, not only the heterogeneity that comes from the usage of different servers inside an HPC facility, but the usage of heterogeneous elements outside of the facility.

Moreover, for large deployments such as the ones of Smart Cities, linear minimization algorithms present scalability problems. This work proposes the usage of non-optimal fast scheduling solutions for the energy optimization problem, by the usage of Satisfiability Modulo Theories (SMT) solvers in a hybrid ambient made of data center HPC servers, PC-like servers and embedded base station systems of WSN infrastructures.

Recently, some enterprises like BrightComputing [23] have begun to develop software tools that allow to allocate tasks both in HPC facilities and in the cloud. This is clearly a response to the need of allocating HPC-workloads in HPC facilities and cloud-workloads in cloud servers. Slurm [24] resource manager will soon provide support for these features too. The application-awareness is thus recognized to be a good way towards energy minimization. However, an accurate application-awareness and energy-optimization allocation algorithm has not yet been proposed.

In this paper, we will be using a modified version of the Slurm resource manager in order to allocate the workload not only in the HPC facility but also in the WSN infrastructure. To do so, we will formally propose and implement a dynamic optimization algorithm that allows the allocation of the workload on runtime inside and outside of the HPC facility.

## 3. Proposed Solution

In this section, the architecture overview of the proposed solution is presented, as well as the requirements that the system has to accomplish. We also explain the different techniques used, and their similarities and dissimilarities when compared to the most common data center allocation scenarios.

### 3.1. Heterogeneous Architecture Overview

**Figure 4.**
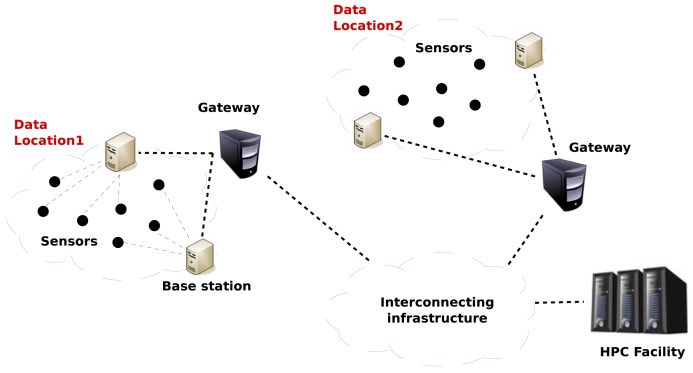
Proposed network topology.

First, in order to develop our solution, we will assume that the aforementioned Smart City architecture is representative. Taking its main elements as a reference, we can state that the services will be deployed over a network with a topology like the one depicted in Figure 4, which is comprised of the following components: (i) sensor nodes; (ii) base stations; (iii) gateways and (iv) HPC servers, organized as follows.

A Wireless Sensor Network (WSN) or a Ubiquitous Sensor Network (USN) is composed of a great number of sensor nodes with very limited resources. A small part of these sensor nodes may have a direct connection to the Internet (via 3G, for example). The majority of them, however, situated in highly dense sensor node areas, will not have a direct connection to the Internet, but will instead transmit their information to a base station. Whereas the sensor node is low-power, battery-operated and has very low resources (typically the sensor nodes have tiny microcontrollers, of tens of MHz of frequency), the base station is usually a microprocessor-based embedded system with higher computational capabilities (working at frequencies of hundreds of MHz). The base stations are often connected via radio to the nodes (e.g., WiFi, Bluetooth, RF, Zigbee), via Ethernet to a gateway or directly to the Internet. They are also usually AC powered. These kind of systems have much more sensor nodes than base stations (in the order of 100–200 nodes per base station).

In some ubiquitous distributed systems, the base station could be the last step between the nodes and the HPC infrastructure that provides and centralizes the services. In the real world, however, and for large deployments such as the ones in Smart Cities, the gateways are responsible for the interaction with the real service provider. These gateways are often PC servers, with higher computing resources than the base stations (e.g., a dual core processor @2 GHz with 2 GB of memory). These PCs can be found either connected to the base stations or not, and usually provide graphical interfaces to end-users for configuration, process data and provide security and trust (see also Figure 1).

It must be noted that, during the normal 24/7 operation of the network, all the sensor nodes, gateways and PC servers will be turned on. The gateways and PC servers might be idle for part of their time; however, they are not turned off. The HPC servers can be turned off or re-used to compute data for other applications. In Table 1 we summarize the approximate parameters of these elements.

The computational demand of the services deployed in the network is often just processed in the HPC infrastructure. In this paper we propose to distribute this computation to the base stations and the gateways of the network. These systems have much lower power consumption than the HPC server and, in their idle times, can be used to process the non-intensive parts of the workload to be executed. Moreover, even though in this paper we will not tackle the cooling costs of the HPC infrastructure, it must be noted that cooling accounts for a 30%–50% of the total energy demand of the HPC infrastructure. Decreasing the computational energy of the HPC servers also decreases the cooling cost. The lower-resource nodes do not need cooling equipment and, thus, the savings are even greater. To distribute the computation towards all the available nodes, we will use the workload allocation and resource managing techniques that are explained in the next subsection.

### 3.2. Workload Allocation and Resource Manager

In order to allocate and manage the computational demands of the services deployed in Smart Cities and ambient intelligence, we will use some scheduling concepts that come from the world of data centers and HPC.

The raw information collected by the WSN sensor nodes will have to be parsed and converted, algorithms will have to be applied and exhaustive processing will be performed to generate useful information. This process comprises the execution of a lot of tasks. As long as the services provided to users do not change, all these tasks, very heterogeneous in nature, will be repeated through time with little changes: the algorithms to be performed each time will be the same, and the only variation will be the data used.

Thus, we can assume that the workload exhibited during a period of time (*i.e.*, one day) is representative of the workload that the WSN and data center facility will have in any other period of time (*i.e.*, the next day). This workload can be understood as a collection of job sets randomly distributed in time. Each job set is composed of a random number parallel tasks without data dependencies; however, the number of different tasks is fixed for all workloads and all the tasks in the job-set are labelled. This means that, when a job-set arrives, the resource manager knows how many tasks of each type it has. As the tasks repeat through time, it will be possible to profile them (in terms of CPU usage, memory usage, *etc.*) and measure the energetic demand (in kWh) of the tasks in the HPC servers, in order to characterize its computing needs. The data used by each of the tasks will be generated in different sensor nodes across the WSN infrastructure. We understand that even though the tasks are the same, the data they use will come from different sensor nodes each time. We assume, however, that the differences in the dataset do not impact the energy profiling of the tasks, as what drives the energy consumption is the algorithm used, not the dataset.

Moreover, once characterization is performed, we will be able to classify the tasks according to their computational demand. This way, tasks exhibiting low (and even medium) computational demands can be allocated to outside the HPC facility.

In order to allocate the tasks to both inside and outside the HPC facility, we will make use of a Resource Manager (Slurm, in this particular case, which is one of the most commonly used). The traditional functional system found in today's data centers comprises: (i) a task scheduler, which queues the tasks in time, deciding their priority of execution; and (ii) a resource manager, which has the knowledge of the available resources of the system and decides where each task is going to be executed.

In our case, we assume that the workload entering the system has already been scheduled by a commercial scheduler, and we implement our solution in the resource manager.

**Figure 5.**
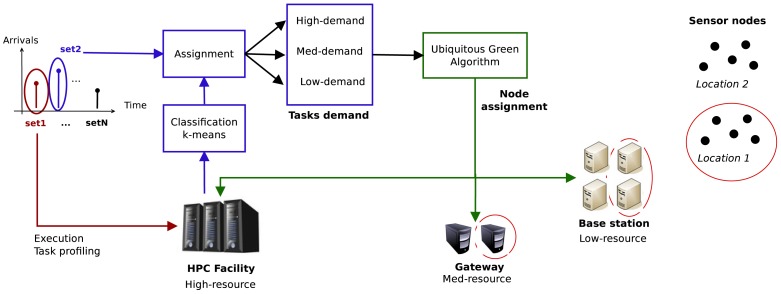
Energy Optimization System.

The complete system is described by Figure 5, and works as follows:

- Cluster creation: Before a service starts, a cluster of machines is created. One of the HPC servers in the facility acts as the Resource Manager (RM). The cluster is composed of an arbitrary number of base stations, gateways and HPC servers. Machines are divided into different partitions according to their resources and are assigned a different location identifier depending on the physical location where they are deployed. Each gateway and base station will manage the tasks whose data comes from sensors in their same (or nearest) location.
- Profiling and classification: When a service is launched, the first job set arriving to the HPC facility is used for profiling and classification purposes. Each different task that composes the job set is sent to a node only in the HPC facility. While executing, the different tasks are profiled to obtain the following parameters: total execution time, memory usage, average CPU load and energy consumption. They are classified by using a naive k-means algorithm in 3 different classes according to their computational demand: high-demand, mid-demand and low-demand. As the tasks of the job-set are labelled, the second and subsequent job-sets will be directly classified into one of these three classes by the allocation algorithm.
- Ubiquitous Green Allocation techniques: the purpose of the allocation is to reduce the energy consumption of the HPC infrastructure while tackling the computational demand of the services in the smart facility. To do so, the allocation algorithm will first identify and classify the new incoming tasks into one of the already-existing groups, which have already been assigned to one of the three different classes. Next, according to the idle resources available, it will try to place high-demand tasks in the HPC servers, mid-demand tasks in gateways and low-demand tasks in base stations. In order to tackle the data locality issues, tasks will be executed as near as possible to where the data they need is generated. This way, data will not have to travel through the WSN infrastructure to the HPC facility.

In Section 4, the Ubiquitous Green Allocation techniques are formally detailed and further explained. These algorithms will be implemented as a new Slurm plug-in and will be executed on runtime each time a new job set arrives.

## 4. Ubiquitous Green Allocation Algorithms

The idea behind this allocation algorithm is to minimize the total energy consumption of the smart infrastructure (by using the processors of the HPC facility and the base stations and gateways of the WSN infrastructure). This goal can be described as follows:

Let us denote by *P* a set of processors and by *T* a set of tasks that must be executed. Each processor *p* belongs to one machine *m*—a machine could be either a server, a gateway or a base station—denoted as *p_m_*, which consumes certain idle power *π_m_*. Every task *t* has a duration and consumes a certain amount of energy depending on the target processor, *σ_tp_* and *e_tp_* respectively. *τ^max^* is the time instant at which all the tasks have been executed. The problem consists in finding the most appropriate allocation of tasks *t* in processors *p*, that minimizes the energy consumption, as expressed in Equation (1). In other words, to globally minimize the energy consumption we have to minimize the sum of (i) the energy variation *e_tp_* that occurs when allocating a particular task *t* in a particular processor *p* and (ii) the energy that machines consume for the fact of being turned on, that is, the idle power they consume multiplied by the complete execution time.

(1) $$\text{Minimize}\;\left\{\sum_{t\in T,p\in P}e_{tp}+\sum_{m\in M}\pi_{m}\cdot \tau^{\text{max}}\right\}$$

However, for our particular case, the allocation step has to take into account several more variables: (i) the number of idle gateways and base stations available when a new job set arrives; (ii) the resources of these nodes and the performance of each task in each node; (iii) the data locality issues; and (iv) the total amount of time to compute the solutions so as to improve performance.

Even though Equation 1 can be solved by means of a linear minimization [10] to obtain energy savings when allocating tasks to heterogeneous nodes, the new restrictions imposed in our case, as well as the need to compute the allocation of a high number of tasks in a high number of nodes, suggest the usage of other solutions rather than an ILP solver. Because of the nature of linear minimization problems, they present good solutions for a relatively small number of nodes. In a deployment aimed to give service to thousands of users, this kind of algorithms does not scale, and it would be better to implement new algorithms that speed-up the allocation process.

Because of its speed and versatility for adding new restrictions, in this work we have decided to implement the solution by means of an SMT solver. Even though this kind of solver does not obtain an optimal solution, we will prove that, if iteratively executed for a small amount of time, it provides solutions that considerably reduce the energy consumed by the facility.

The proposed solution can be implemented in a two-step iterative algorithm:

- To calculate the best type of node for each task: this step comprises the assignment of different types of tasks to different types of processors. Given the resulting 3 classes of the task classification step, we have to match different cpu-demand tasks to resources. The algorithm will take into account the place where the dataset needed to perform the task was generated, as well as the amount of resources near the data generators. That is, depending on the number of processors of each type (base station, gateways, HPC), the number of tasks of each type, and the place where data was generated, we will get and idea of where should each type of task be executed. The general constraint here will be that: (i) a low-resource node can only execute a subset (or the whole set) or low-demand tasks; (ii) a medium-resource node can execute a subset of low-demand and mid-demand tasks; and (iii) a high-resource node can execute all tasks. The SMT solver decides which subset of tasks from the allowed ones does each node execute.
- To assign tasks to nodes: The second step consists in a greedy solution that tries to allocate the maximum number of tasks (without exceeding a maximum time) in the nearest lowest-resource nodes first—which are the best from the energy-efficiency point of view. That is, it will try to allocate first the low-demand tasks in Base Stations nodes. The SMT solver checks whether the allocation is possible and obtains a solution. If the conditions are satisfiable, it proceeds to allocate mid-demand tasks in gateway nodes. If they are not, it allocates less tasks in base station nodes, and allocates the remaining tasks in medium-resource nodes. In order to decide which tasks should remain in low-resource nodes or be migrated to mid-resource nodes, we again make use of the algorithm in the first step. All the tasks than can neither be executed in low-resource nodes nor in medium-resource nodes are executed in the HPC facility.

**Figure 6.**
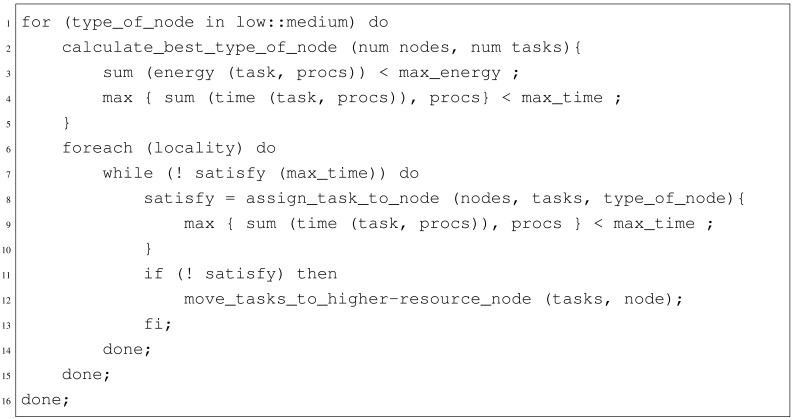
SMT Solver Algorithm pseudocode.

The pseudo-code for the complete algorithm is given in Figure 6.

We choose the maximum time as the maximum for a similar scheduling in the HPC infrastructure—that is, the case in which all tasks are allocated in high-resource nodes. That time is reduced by using the SMT solver to place low and medium demand tasks in low and medium resource nodes. It must be noted that locality is managed in such a way that, if the algorithm is unable to allocate a task of, e.g., locality 1 into either the low or the medium-resource nodes, it will allocate the task into the HPC facility, not into an element belonging to locality 2. This way, the algorithm minimizes the communication energy between nodes as well. The aforementioned algorithm will have to be executed each time a new job set arrives, in order to allocate the workload.

## 5. Results

In this section we present the results obtained when applying the classification and allocation techniques proposed in this paper. These techniques have been tested in the three different real machines that have acted as the different elements of the smart environment. Their parameters are shown in Table 2.

**Figure 7.**
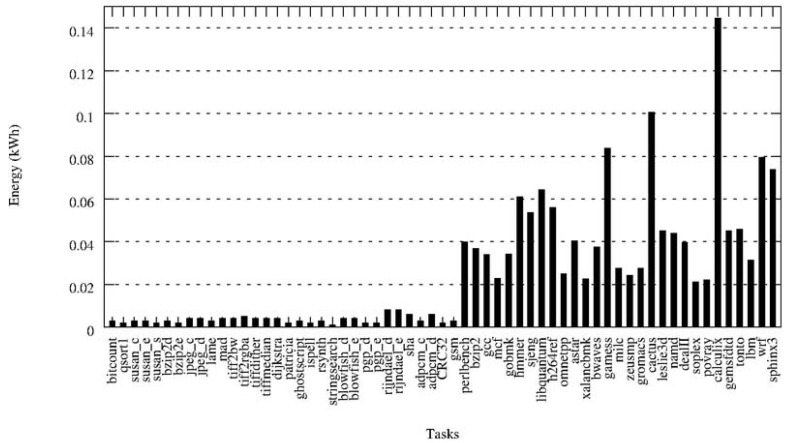
Energy profiling of the tasks in the Intel processor.

**Figure 8.**
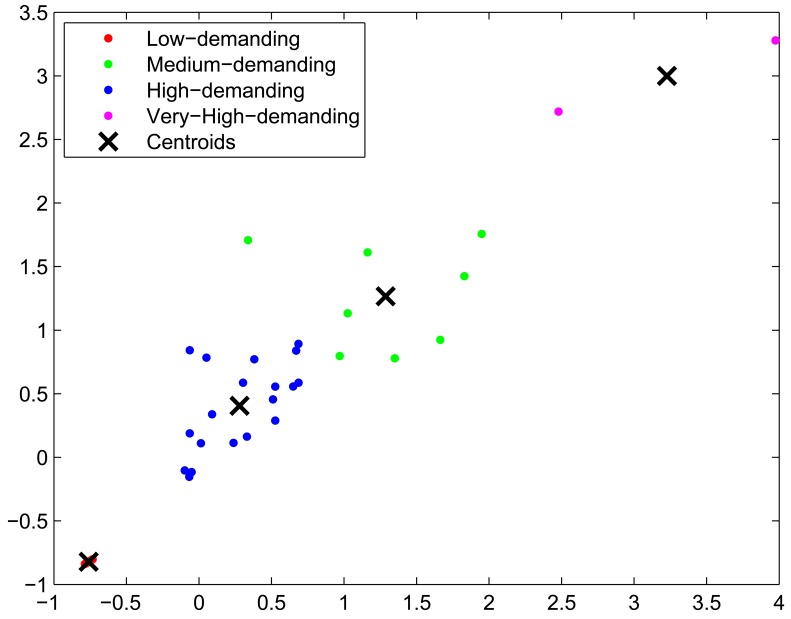
Clustering results. Projection over energy and time axis.

**Figure 9.**
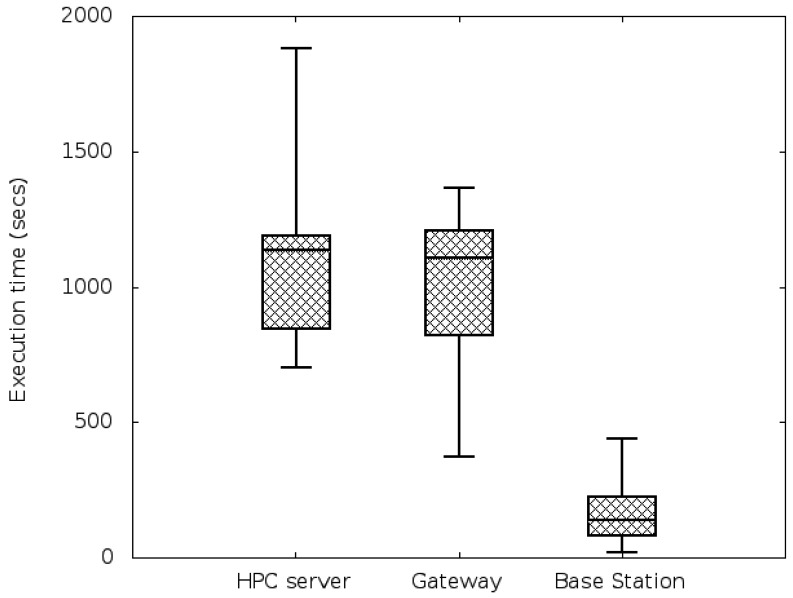
Execution time (in seconds) for tasks in their class.

As we could not try our solution in a real smart infrastructure with thousands of sensor nodes nor in a real HPC facility, we had to use the real energy consumption and timing values of these three elements to simulate most of the behavior of the real system. The steps followed in order to evaluate our methodology are the following:

- Generation of a network that contains the three different types of elements. We have worked with one simulated network of 10,000 sensor nodes, 200 base station nodes, 15 gateways and 10 HPC servers (with 8 cores each). The elements are evenly split into 3 different localities, so that the number of all the elements is approximately the same in all the localities—e.g., locality 1 will have 3,300 sensor nodes, 30 base station and 5 gateways. If a task is executed in the HPC facility, its dataset will travel from the sensors that generated it, directly to the nearest base station and gateway in their locality in order to get to the HPC facility—*i.e.*, data will make 3 hops. If a task is executed in a Base Station, then data will only travel 1 hop. As it can be seen, the unity to measure the locality is the number of hops data has to travel from the source—the sensor node—to the destination —the node where it is used for computation.
- Generation of a synthetic heterogeneous workload that emulates the workload of a real service in a smart environment: to do so, we have combined cpu-intensive and non-cpu-intensive tasks into the job sets that compose the workload. We have used all the tasks from the SPEC CPU 2006 benchmark [26] (which are very computationally demanding) and from the Collective Benchmark [27]. This means a total of 60 different types of tasks. A synthetic random workload of 1,500 tasks, randomly split in different job sets of 150, 200, 250 or 300 tasks and with random arrival times of 10, 20 or 30 minutes, has been generated.
- Profiling of the tasks of the first job set in the IntelXeon machine: as explained in Section 3.2, we use the first job set of the workload to profile the tasks in the HPC facility. The profiling step gathers information about the following features for each task: average CPU usage, memory used, time needed to complete execution and energy. On Figure 7 the results for the energy profiling of the tasks are shown. The Y-axis represents the energy variation (in kWh) when allocating a certain task in a certain processor—that is, the values of *e_tp_* for *p* being an Intel processor. This Figure lets us deduce intuitively the three different types of tasks: the low-demand tasks consume very little energy, the medium-demand tasks consume a little more, while there are other tasks that comparatively consume a lot of energy. However, making this assumption only with the energy results and without paying attention to other characteristics such as the CPU-usage would be a naive approximation. Therefore, in the next step a clustering that takes into account all the features is performed.
- Task classification of the first and the subsequent job-sets: using all the characteristics obtained during the profiling step, a naive k-means algorithm splits the different tasks into three different classes, according to their computational demands. A projection of the resulting clustering on the energy and time axis is shown in Figure 8. As expected, the low-energy tasks (which also have low CPU-demand) are assigned to low-demand classes and the CPU-intensive tasks are divided into mid-demand and high-demand classes. According to this clustering and because all the tasks of the job set are labelled, each task will be automatically assigned to one of the classes. As in this paper we are trying to assign low-demand tasks to low-resource nodes, mid-demand tasks to midresource nodes and high-demand tasks to the HPC facility by means of the allocation algorithm, a good clustering will be one that splits tasks such that their execution time in each processor is coherent; that is, the allocated task properly adapts to the resources it has been assigned to. In order to validate our clustering, we execute the tasks in the processors where they are classified and we measure their execution time. Results are shown in Figure 9. As it can be seen, tasks are executed within proper time limits, and there are not low-resource tasks that need too much execution time. Because the Base Stations consume less power than the Gateways, and the Gateways less than the HPC servers, we can also conclude that the energy graphic will have the same shape as the time graphic of Figure 9.
- Ubiquitous Green Allocation: The allocation algorithm is validated by using Slurm resource manager to simulate the whole workload allocation and distribution—that is, the 1,500 tasks. Slurm can be run on multiple-slurmd mode to emulate a cluster of machines, setting the task affinity to core. This means that Slurm will bind each task to one and only one core, and a machine will execute as many tasks in parallel as available cores. We have programmed a Slurm plug-in that allows us to change the allocation policy. This will let us compare Slurm default allocation (which consists of a round-robin assignment policy) with the allocation proposed by our SMT solver algorithm.
- Energy and time savings calculation: Once all the workload has been executed, we calculate the total energy consumed by the allocation and compare it to the energy consumed if the workload had been executed only in the HPC facility. In this calculation we take into account both the savings obtained by executing tasks in less energy-consuming nodes, and the energy savings that come from the decrease in execution time and thus, in idle energy of HPC servers. We also provide data on the communication savings, given as the number of hops for the datasets going through the WSN infrastructure.

Table 3 shows the results, in terms of the energy used to execute tasks, the total energy consumed (energy to execute tasks plus idle energy of the HPC machines for the fact of being turned on), execution time of the allocation for different configurations of HPC servers (HPC), Base stations (BS) and gateways (GW), and the average number of hops for the datasets (that is, the communication energy). We have considered that BSs are idle and available to compute during a 25% of their time; GWs are idle and available during a 50% of their time, and HPC servers dedicated for this computations, and thus can be used at 100%.

The first row of the table presents the results for the state-of-the-art allocation: only the resources of the HPC facility are used for computation, and the resource manager Slurm uses its default round-robin allocation algorithm. It must be noted that the average number of hops for a dataset is 3, because the information gathered by the sensors has to travel to the HPC facility going through a BS and a GW. Our goal is, taking this case as the reference, to minimize energy and maximize performance.

The second, third and fourth rows show the results for our SMT solver algorithm given different combinations of the resources to be used—*i.e.*, a different amount of GW and BS used for computation. In this case, the resource manager uses the results of the SMT solver to perform the allocation.

As it can be seen, with the usage of the proposed techniques and algorithms we can achieve from 10% up to a 40% speed-up in the execution time of the workload, which is also translated into energy savings in the idle energy consumed by the machines—as they can be turned off, or simply used to process other services. The total energy used to execute the tasks also decreases in a 48% for the best case. It must be noted that this results are highly dependent on the number and the combination of different types of nodes that form the cluster, as well as on the percentage on non-cpu-intensive tasks that make up the workload. In this sense, if our workload has a large number of non-cpu-intensive tasks—approximately 50%, as it is in this case—then we will obtain very good results by increasing the number of low and medium resource machines. On the other hand, should the workload consist only of cpu-intensive tasks, the benefits would be much less.

As for the communication infrastructure, even though we have neither calculated nor taken into account the savings, it is clear that tackling with locality allows us to reduce dataset traffic through the infrastructure. If a particular task that needs a particular dataset is executed in a BS or a GW near the source, data does not have to travel to the HPC facility, and communication costs are reduced.

These solutions have been computed by using the Yices SMT Solver [28]. As for the execution time, it must be said that the maximum execution time needed to allocate a jobset was 4 minutes. As most of the tasks have higher execution times, this is a feasible solution, which can be used as a Slurm plug-in to be executed on runtime, during the normal execution of the workload. As a drawback, a spare core will be needed in order to calculate the task allocation. However, this does not have an impact an energy consumption. One core of the HPC facility working at its maximum power consumption for at most 4 minutes each time a new job-set arrives means a worst-case energy consumption of less than 1 kWh for the whole workload.

## 6. Conclusions

This paper proposes novel energy management techniques to tackle the computational needs of smart cities and ambient intelligence applications, by making use of energy-minimization workload assignment policies. These techniques are inspired by the solutions that come from the world of data centers. They use application-awareness and heterogeneity in order to assign low-demand and mid-demand computational tasks to idle nodes with low and medium resources in the WSN infrastructure, instead of executing them in the HPC infrastructure. The proposed solution uses SMT solvers to generate energy-efficient assignments that take into account several variables such as maximum execution time and data locality. The results prove that this kind of non-optimal assignment can increase the energy savings of the smart infrastructure up to a 40%, mainly because of the savings that come from decreased execution times. Also, the usage of this kind of algorithms allows the implementation of energy-efficient task assignments in large sensor deployments.

Future work will focus on the development of more accurate and efficient SMT solver algorithms, which contemplate more constraints of the problem, as well as on the usage of real smart environment applications, such as healthcare services. Also, an accurate comparison between the performance of ILP minimizations and SMT solvers for different sizes of the WSN deployment is envisioned. In the near future a simulation framework will be developed that integrates all the components together—HPC infrastructure, WSNs, communications and Slurm RM—in order to evaluate more accurately the beneficial impact of the data locality on energy savings and performance.

## Figures and Tables

**Table 1.** Network components.

**Element**	**Processor**	**Frequency**	**RAM**	**Idle Power**	**Max. Power**
Sensor node	Microcontroller	50 MHz	256 KB	<30 mW (<3 mV sleep)	<100 mW
Base station	Embedded *μ*P	500 MHz	256 MB	3 W	5 W
Gateway	Dual-core	2 GHz	1 GB	75 W	150 W
HPC server	2 × Quad-core	2.6 GHz	8 GB	150 W	750 W

**Table 2.** Parameters for simulated sensor network.

**Element**	**Machine**	**Frequency**	**RAM**	**Idle Power**	**Max. Power**
Base station	MPC8315E Zignus [25]	400 MHz	256 MB	2.4 W	3 W
Gateway	AMD Athlon64	2.5 GHz (dual-core)	1 GB	75 W	125 W
HPC server	Fujitsu RX300 S6	2.6 GHz (2 × Quad-core)	8 GB	150 W	750 W

**Table 3.** Green Allocation algorithm results.

**Sensors**	**HPC**	**BS**	**GW**	**Energy (kWh)**	**Total (kWh)**	**Time (h)**	**Avg hops**
5000	10	0	0	37	87	9.4	3
5000	10	100	10	34	79	8.5	2.4
10,000	10	200	10	34	69	6.6	2.1
10,000	10	200	15	30	45	5.6	2.0
